# Supplementary material for: Combined Pharmacological Conditioning of Endothelial Cells for Improved Vascular Graft Endothelialization
Source: Int J Mol Sci. 2025 Jul 25;26(15):7183. doi: 10.3390/ijms26157183 (PMC12346220; doi:10.3390/ijms26157183)
Supplement: Supplementary file 1 [file ijms-26-07183-s001.zip › ijms-3699628-supplementary.pdf]

Supplemental Table S1, Figures S1 and S2

Table S1. Primer sequences for qPCR

Gene	Primer
ACTB	Forward: TTGTTACAGGAAGTCCCTTGCC
	Reverse: ATGCTATCACCTCCCCTGTGTG
ZO-1	Forward: AAGAGATGAACGGGCTACGC
	Reverse: GTTGAGACAGCCCCAGGTTT
CLDN1	Forward: TTCATTCTCGCCTTCCTGGG
	Reverse: GACTTTGCACTGGATCTGCC
CLDN5	Forward: CGCGCACGGATTGGC
	Reverse: GTACCCTCTTTGAAGGTTCGGG
JAM-A	Forward: TGTGCCTACTCGGGCTTTTC
	Reverse: TGGAGGCACTGTGATCTTGT
JAM-B	Forward: ACAAGAAGTGATGCGGGGAA
	Reverse: GGAACTGCTGGAGCCACTAA
JAM-C	Forward: ATGATGTACCACTGCCCACG
	Reverse: CATCTCCTGCTCCTCACACC
VWF	Forward: TTGGGGACTTCCAGAATGGC
	Reverse: GACTCTTTGGTCCCCCTGTG
F3	Forward: CCTGGAGACAAACCTCGGAC
	Reverse: CCCGGAGGCTTAGGAAAGTG
ANGPT1	Forward: GCAGCCTGATCTTACACGGT
	Reverse: GCCACAAGCATCAAACCACC
ANGPT2	Forward: GCATCAGCCAACCAGGAAATG
	Reverse: GCCTGAGCCTTTCCAGTAGT
AKT1	Forward: GGACAAGGACGGGCACATTA
	Reverse: CGACCGCACATCATCTCGTA
RHOA	Forward: ATTGTTGGTGATGGAGCCTGT
	Reverse: CCATCCACCTCGATATCTGCC
NFKB1	Forward: TGGTGGAGTCTGGGAAGGAT
	Reverse: TCCTCCGAAGCTGGACAAAC
NOTCH1	Forward: TGTGCCAGTACGATGTGGAC
	Reverse: GGTGTAAGTGTTGGGTCCGT

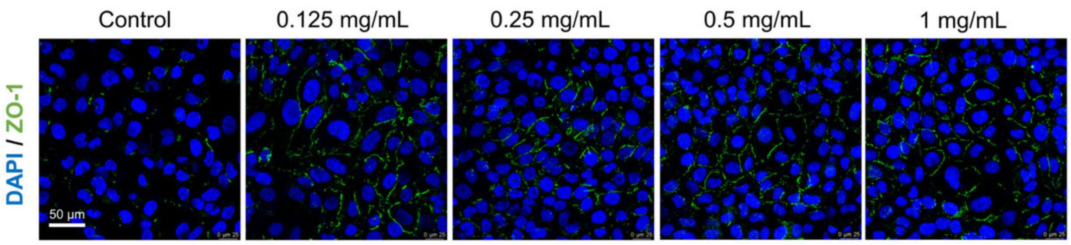

Figure S1. ZO-1 expression in HUVECs treated with gradient concentrations of pCPT-cAMP.

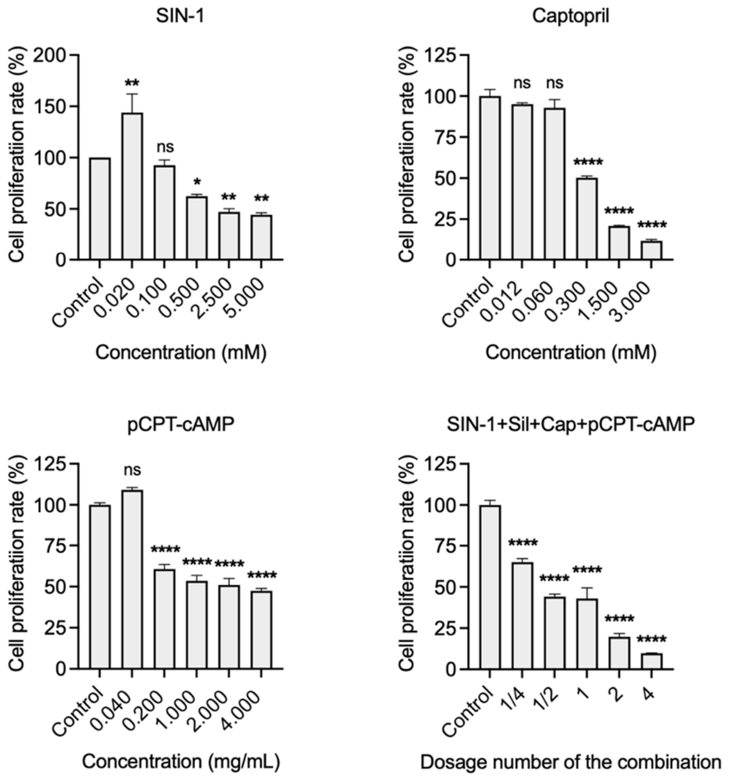

Figure S2. Cell Toxicity of the four-component combination and individual drugs. One dosage of the combination consists of 0.1 mM SIN-1, 0.3 mM Captopril. 0.01 mM Sildenafil and 1 mg/mL pCPT-cAMP. Data are presented as mean ± SEM (n = 3 biological replicates; $*p < 0.05$, $**p < 0.01$, $****p < 0.0001$ vs. control). Data were analyzed using Dunnett's multiple comparisons test.
